# Research on Cloud-Edge-End Collaborative Computing Offloading Strategy in the Internet of Vehicles Based on the M-TSA Algorithm

**DOI:** 10.3390/s23104682

**Published:** 2023-05-12

**Authors:** Qiliang Xu, Guo Zhang, Jianping Wang

**Affiliations:** Faculty of Information Engineering and Automation, Kunming University of Science and Technology, Kunming 650500, China; xu_qiliang@stu.kust.edu.cn (Q.X.); wjp@kust.edu.cn (J.W.)

**Keywords:** Internet of Vehicles, collaborative computing, computational offloading, M-TSA

## Abstract

In the Internet of Vehicles scenario, the in-vehicle terminal cannot meet the requirements of computing tasks in terms of delay and energy consumption; the introduction of cloud computing and MEC is an effective way to solve the above problem. The in-vehicle terminal requires a high task processing delay, and due to the high delay of cloud computing to upload computing tasks to the cloud, the MEC server has limited computing resources, which will increase the task processing delay when there are more tasks. To solve the above problems, a vehicle computing network based on cloud-edge-end collaborative computing is proposed, in which cloud servers, edge servers, service vehicles, and task vehicles themselves can provide computing services. A model of the cloud-edge-end collaborative computing system for the Internet of Vehicles is constructed, and a computational offloading strategy problem is given. Then, a computational offloading strategy based on the M-TSA algorithm and combined with task prioritization and computational offloading node prediction is proposed. Finally, comparative experiments are conducted under task instances simulating real road vehicle conditions to demonstrate the superiority of our network, where our offloading strategy significantly improves the utility of task offloading and reduces offloading delay and energy consumption.

## 1. Introduction

With the development of 5G technology and smart connected cars, cars have become equipped with stronger computing and storage capabilities, as well as information collection and communication capabilities, and many new in-vehicle applications have emerged. Although these applications can enhance the user experience and improve driving safety, such as AI-based applications, virtual reality, intelligent assisted driving, image navigation, and entertainment applications, they all have high requirements for computing and storage resources and are sensitive to latency. The computational demand for the Internet of Vehicles has thus boomed [1,2,3], and the limited computational storage resources of in-vehicle terminals cannot meet the resource demand of computational tasks with high complexity, data density, and delay sensitivity [4].

The introduction of cloud computing and Mobile Edge Computing (MEC) into the Internet of Vehicles is an effective way to solve the above problems, but the in-vehicle terminals have high requirements for task processing delay because the cloud computing uploads computing tasks to the cloud with high delay. Furthermore, the computing and storage resources of edge computing servers in MEC are limited, and more tasks will increase the task queuing delay at the server. Therefore, collaborative central cloud, edge cloud, and vehicle cloud computing provide better computing services for task vehicles. The vehicle cloud is a resource of simultaneously empty idle vehicle terminals [5].

Initial progress has been made in the research of collaborative computing for the Internet of Vehicles scenario, where the computational offloading decision is the core research point, and the key is to find the optimal offloading decision to improve the computational efficiency and reduce the computational cost. Unfortunately, the computational offloading problem of collaborative computing in the Internet of Vehicles scenario is a mixed integer nonlinear programming (MINP) problem, which is difficult to solve directly using traditional mathematical methods. Although many scholars have studied computational offloading strategies for the Internet of Vehicles scenario, there is no popular general solution method yet. Based on the above, we study the offloading strategy of collaborative computation at the cloud-edge-end of the connected vehicle scenario with real road conditions and vehicle motion and fully adopt the intelligent swarm optimization algorithm to solve the problem and comprehensively optimize the computational delay and energy consumption. The main contributions of this work can be summarized as follows:

(1) A three-layer architecture of the central cloud, edge cloud, and vehicle cloud is proposed as a cloud-edge-end collaborative computing system model with the task offloading strategy problem in an Internet of Vehicles scenario.

(2) A Multi-strategy collaboration-Tunicate Swarm Optimization Algorithm (M-TSA) introduces a memory learning strategy, a Levy flight strategy, and an adaptive dynamic weighting strategy on the basis of the standard TSA algorithm, has stronger global optimization seeking capability, and is proposed for multi-objective optimization of offloading delay, energy consumption, and task offloading utility of cloud-edge-end collaborative computing systems.

(3) To address the offloading strategy problem presented in (1), a computational offloading strategy based on the M-TSA algorithm and combined with task prioritization and computational offloading node prediction is proposed to significantly improve the system offloading utility and reduce the computational offloading delay and energy consumption of the system by taking into account the vehicle motion characteristics and task time delay sensitivity.

## 2. Related Work

### 2.1. Edge Computing Offloading

Many experts and scholars currently have relatively mature research work on computational offloading strategies, mainly optimizing or jointly optimizing metrics such as time delay and energy consumption. There are mainly computational offloading frameworks based on mathematical models [6,7], computational offloading schemes based on intelligent optimization algorithms such as genetic algorithms [8,9,10], and whale optimization [11]. Offloading strategies for collaborative computing have also been studied. Dai et al. [12] designed a probabilistic computational offloading algorithm for cloud-edge collaborative computing and verified its superiority in reducing task delay in a wide range of scenarios. Abbasi et al. [13] addressed the problem of allocating workloads in a fog cloud scenario and proposed a trade-off between task processing energy consumption and delay for the NSGA-II algorithm to solve this multi-objective model, and they experimentally showed that both energy consumption and delay were significantly reduced. Huang et al. [14] studied an optimal offloading scheme considering energy minimization, which addresses the relationship between energy efficiency and performance in mobile The relationship between energy efficiency and performance in mobile edge computing systems was investigated. The results showed that this scheme is better than other offloading methods. Zhao et al. [15] explored the collaborative computation offloading problem in a MEC system with multiple users in a heterogeneous cloud system. Based on dynamic planning, an energy consumption minimization algorithm with joint bandwidth and computational resource allocation was proposed, and the simulation results showed a reduction in energy consumption for mobile devices. Ramtin et al. [16] proposed an offloading scheme based on inter-device collaboration for jointly optimizing energy consumption and delay in edge computing, applying the maximum matching and minimum cost graph algorithms to derive a reasonable offloading scheme, and the results showed a reduction in energy consumption and delay. Fu et al. [17]—taking into account the effects of changing network conditions and wireless channel constraints—proposed an improved firefly swarm algorithm that optimizes computation offloading and resource allocation to reduce computation system latency and energy consumption. Li et al. [8] proposed a genetic-algorithm-based two-stage heuristic for joint computation offloading and resource allocation in multi-user and multi-server scenarios, and they proved the effectiveness of their algorithm for reducing terminal energy consumption. Su et al. [18] proposed a resource deployment and task scheduling algorithm based on task prediction and Pareto optimization. The user service quality and system service effect were significantly improved.

### 2.2. Collaborative Computing Offloading under the Internet of Vehicles

Preliminary research has also been conducted on collaborative computational offloading strategies for special scenarios of the Internet of Vehicles. Zhao et al. [7] proposed a joint optimization scheme for computational offloading and resource allocation based on a mathematical model to effectively improve the system utility and computation time of MEC in scenarios with insufficient computational resources; however, their algorithm is not general. Xu et al. [19] proposed an adaptive multi-objective evolution (ACOM) offloading method for IoCV scenarios with the introduction of 5G, which reduces the task offloading delay but does not consider the impact of vehicle mobility characteristics. Song et al. [20] constructed a unidirectional highway model under which edge servers and vehicle servers work together, described a safe switching interaction protocol while the vehicle is moving, and reduced offloading energy consumption and delay. Zhang et al. [21] constructed an SDN-assisted MEC network architecture for vehicle networks and proposed a joint task offloading and resource allocation strategy that can effectively reduce system overhead. Zhu et al. [22] designed a cloud-edge collaborative-based vehicular computing network architecture, proposed an offloading strategy scheme based on an improved multi-objective optimization immune algorithm, and verified the effectiveness of the algorithm. In the research of Shen et al. [23], a hybrid genetic algorithm (HHGA) task offloading strategy with a hill-climbing operator was proposed for mobile edge computing with on-street parking collaboration in the Internet of Vehicles to reduce the delay and energy consumption of computational tasks. Lastly, Su et al. [24] proposed an improved sparrow-algorithm-based computational offloading decision for cloud-edge collaborative computing to fully optimize task delay and energy consumption.

In summary, the research on edge computing offloading strategies is more mature, but the research on collaborative computing offloading for special scenarios such as the Internet of Vehicles is lacking, and the impact of hidden vehicle movement characteristics, task priority offloading, real road traffic conditions, and other factors, as well as the problem of ignoring idle vehicle terminal resources, are seldom considered in the research on computing offloading for Internet of Vehicles scenarios. To address the above problems, we will study the cloud-edge-end collaborative computational offloading strategy under the real road traffic condition and vehicle movement in the Internet of Vehicles scenario.

## 3. Cloud-Edge-End Collaborative Computing System Model in the Internet of Vehicles Scenario

**Figure 1:**
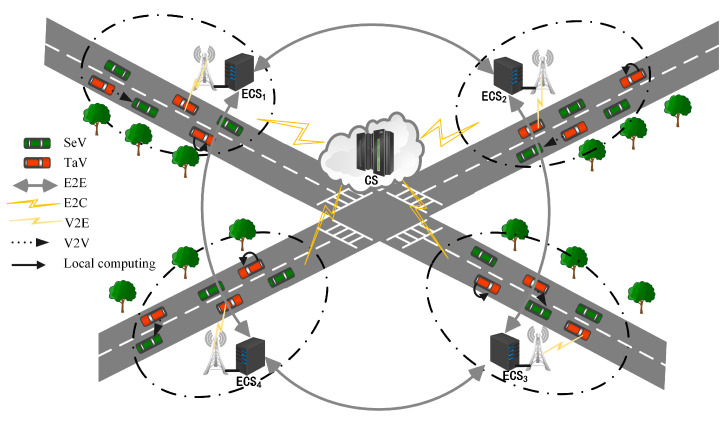
Cloud-edge-end collaborative computing network diagram in the Internet of Vehicles scenario.

The cloud-edge-end collaborative computing network in the Internet of Vehicles scenario described in this system consists of vehicles, base stations (BS), Edge Computing Servers (ECS), and Cloud Servers (CS). As shown in Figure 1, in a two-way straight-road scenario, many base stations equipped with edge servers are evenly deployed on the roadside, and their communication coverage radius is *L*. The vehicles and ECS in the communication area of BS are called an edge computing domain. There are two types of vehicles in an edge computing domain: one is task vehicles (TaV) that generate computational tasks; the other is service vehicles (SeV) that have many available computational resources and can provide computational services to the outside world. The set of edge servers is denoted as Es={1,…m,…M}, and the sets of TaV and SeV in the ${\mathrm{ECS}}_{m}$ edge computing domain are denoted as ${Ta}_{m}=\left\{1,\ldots i_{m},\ldots I_{m}\right\}$, ${Se}_{m}=\left\{1,\ldots j_{m},\ldots ,J_{m}\right\}$. For the convenience of the following formulation, the service vehicles SeV, edge servers, and cloud servers providing service computing are collectively referred to as service computing nodes in this system and are denoted as $N=\left\{0,1,\ldots m,\ldots ,M,M+1,\ldots M+j_{m},\ldots ,M+J_{m}\right\}$. To efficiently utilize the spectrum, this system considers an OFDMA-based wireless network that connects the ECS with the task vehicle TaV and the service vehicle SeV to form a star topology, where each vehicle can communicate with the ECS in one leap point; wired connections are used between adjacent edge servers and between the ECS and the CS. In this computing network, ECS is the manager of the computing domain and is responsible for the scheduling and allocation of all tasks. At the beginning of each time slot, each vehicle in the computing domain uploads task information and computing resource information to the edge server. There are vehicles with many available computing resources, which are what we call SeV. ECS aggregates the computing tasks and the resources of service vehicles through the intelligent scheduling of tasks, which can provide higher-quality computing services to the task vehicles at the end of the network. The parameters used in this paper are listed in Table 1.

There are mainly vertical and horizontal collaborative computing methods for the vehicles described in this system model, and there are various servers that can provide computing offload services for the task vehicles in this model, namely, cloud servers, edge servers, terminal devices of the service vehicles, and terminal devices of the task vehicles themselves. Through the intelligent scheduling of tasks, the effective utilization of global resources can be realized, and the task vehicles at the end of the network can be provided with a more high-quality computing offload service. Vertical and horizontal collaboration are differentiated as follows:

(1) Vertical collaboration: Comprised of the vehicle cloud, edge cloud, and central cloud, the three-layer Internet of Vehicles edge computing architecture provides multiple offload mode options for resource-constrained task vehicles. Thus, task vehicles can choose to process their tasks locally according to the actual situation or offload tasks to neighboring service vehicles, edge servers, and cloud servers to achieve task processing.

(2) Horizontal collaboration: The distribution of resources in the time dimension of edge servers often shows variability. Lightly loaded edge servers may cause waste due to unutilized resources, while overloaded servers may affect the normal processing of tasks due to insufficient resources. Therefore, cross-domain edge collaborative computing can be used to improve the efficiency of system resource utilization and enhance the offloading utility of tasks.

### 3.1. Vehicle Motion Model

**Figure 2:**
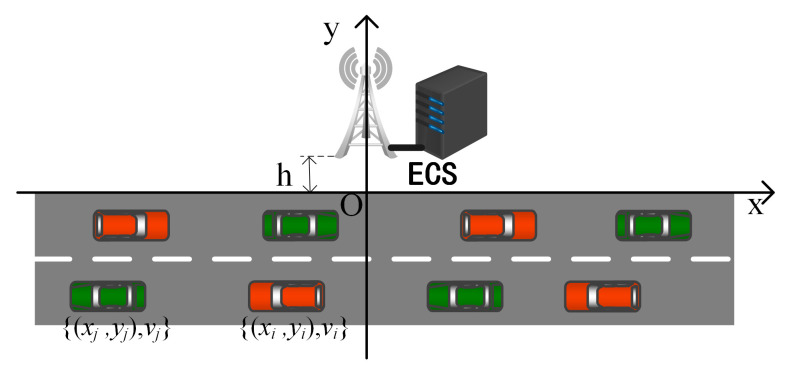
Vehicle movement diagram.

The system uses a two-dimensional coordinate system to model the motion process of the vehicle, as shown in Figure 2, denoting the BS side of the road as the *x*-axis and the vertical line of BS as the *y*-axis, thus assuming that the coordinates of BS as (0,h), where *h* is the linear distance between BS and the road. The ${\mathrm{TaV}}_{i}$ movement pattern can be represented by a binary group as $\left\{\left(x_{i},y_{i}\right),v_{i}\right\}$, whereby $\left(x_{i},y_{i}\right)$ is ${\mathrm{TaV}}_{i}$ the starting position and $v_{i}$ is the ${\mathrm{TaV}}_{i}$ travel speed. Assuming that the right is the positive direction, a positive sign of $v_{i}$ indicates that ${\mathrm{TaV}}_{i}$ rightward travel, and the negative sign of $v_{i}$ indicates that ${\mathrm{TaV}}_{i}$ travels to the left. Similarly, the ${\mathrm{SeV}}_{j}$ movement pattern is represented by the binary group $\left\{\left(x_{j},y_{j}\right),v_{j}\right\}$.The standard lane width of the road is 3 m. This system assumes that all vehicles travel in the middle of the lane, i.e., the vehicle vertical coordinate is y∈{−1.5,−4.5}.

This system establishes a vehicle movement model constrained by speed and distance to simulate the real road vehicle driving environment. Since the calculated offloading time of the vehicle Δt is very small, it is assumed that the vehicle maintains a uniform speed during the time Δt, that is, $v_{i}\left(t+\Delta t\right)=v_{i}\left(t\right)$, $v_{i}\left(t\right)$ denotes the vehicle $vh_{i}$ velocity at *t* moment. There are two constraints in this model:

(1) Speed constraint: Because there is a speed limit on the real road, the speed of each vehicle must be maintained in a range, i.e., $v_{i}\in \left[v_{\min\;},v_{\max\;}\right]$.

(2) Distance constraint: Two vehicles in the same lane, $vh_{i}$ at position $x_{i}$ and $vh_{j}$ at position $x_{j}$, need to satisfy $\left|x_{i}-x_{j}\right|\in \left[l_{\min},l_{\max}\right]$. $l_{\min}$ indicates the minimum distance between two vehicles driving continuously on the same lane, also known as the safety distance; if the distance between two cars is too small, it will increase the risk of traffic accidents. $l_{\max}$ denotes the maximum distance between two vehicles driving continuously on the same lane; if the distance between the two vehicles is too large, it will be a waste of traffic resources. Therefore, the distance between two vehicles must be kept within a reasonable range.

### 3.2. Computational Model

The computational task for a single time slot of each task vehicle ${\mathrm{TaV}}_{i}\in Ta$ is considered in this system, denoted as $T_{i}$, which is the smallest task and cannot be divided into subtasks. Each task vehicle ${\mathrm{TaV}}_{i}$ generates a computational task $T_{i}$, which is represented by three parameters $\left\{b_{i},c_{i},t_{i}^{\max\;}\right\}$, where $b_{i}$ (bit) is the amount of data required to complete the task, that is, the amount of input data required for the computational task execution to be transmitted from the task vehicle local device to the service computation node; $c_{i}$ (cycles) means the amount of computation to complete the task; and $t_{i}^{\max}$ refers to the maximum delay limit of the computation task, determined by the task type. Each task can be executed locally in the task vehicle or offloaded to the service vehicle ${\mathrm{SeV}}_{j}$, the ECS, or the CS. Each service compute node has independent storage resources $B_{n}$ and compute resources $C_{n}$. The task vehicle saves energy and task processing time by offloading the compute tasks to the service compute node; however, the amount of compute task input data sent to complete the task in the compute task offload adds additional time and energy consumption.

This section defines the task offloading variables, and the equation includes the upstream scheduling as follows: $\left\{a_{i,j},i\in Ta,j\in N\right\}$, where $a_{i,j}=1$ means that the task vehicle from ${\mathrm{TaV}}_{i}$ of the task $T_{i}$ is offloaded to the service compute node; otherwise, $a_{i,j}=0$. Since each task can be executed locally or offloaded to up to one service compute node, a feasible offloading strategy must satisfy the following constraints:

(1) $$\sum_{j\in N}a_{i,j}\leq 1,\forall i\in Ta$$

The location of the calculation task $T_{i}$ generated by the task vehicle ${\mathrm{TaV}}_{i}$ is as follows:

(2) $$a_{i,j}=\left\{\begin{array}{c} 0,j\in N,T_{i}\;\mathrm{Local}\;\mathrm{computing}\\ 1,j=0,\;T_{i}\;\mathrm{Offloading}\;\mathrm{to}\;\mathrm{CS}\;\\ 1,0<j\leq K,\;T_{j}\;\mathrm{Offloading}\;\mathrm{to}\;\mathrm{ECS}\;\\ 1,j>K,\;T_{i}\;\mathrm{Offloading}\;\mathrm{to}\;\mathrm{SeV} \end{array}\right.$$

For each task vehicle ${\mathrm{TaV}}_{i}\in Ta$ generated, due to limited computing resources, some of the computation tasks need to be transferred to the SeV, the ECS, or the CS, which then performs the computation. Since the SeV computation storage resources are limited, in this study, SeV considers single-task computation and does not create a task cache. In this system, a task queue model of the task buffer of ECS and CS is established, and Q(t+1) denotes the accumulated tasks at the moment t+1, that is,

(3) Q(t+1)=max{Q(t)−Φ(t),0}+D(t)

where Φ(t) is the size of the computational task that leaves the task buffer of ECS at time slot *t*, i.e., the task for which ECS completes the computation, and D(t) is the size of the computational tasks that are offloaded to the task buffer of the ECS by the task vehicle at time slot *t*.

### 3.3. Delay Model

Once the task vehicle ${\mathrm{TaV}}_{i}$’s calculation of task $T_{i}$ processing is complete, the resulting delay time $t_{i}$ includes: i. upload delay $t^{\mathrm{up}}$ (s)—the time to transmit the input on the uplink to the service node *N*; ii. cache delay $t^{q}$ (s)—the queuing time in the task buffer; iii. computation delay $t^{\mathrm{exe}}$ (s)—the task computation processing time; and iv. the time to transmit the output on the downlink from the service compute node *N* to the task vehicle ${\mathrm{TaV}}_{i}$. These are described in more detail as follows:

(1) Upload delay: When the transmission rate of the communication between the computing nodes and the amount of task input data $b_{i}$ are related, then the upload delay $t^{\mathrm{up}}$ of task $T_{i}$ is

(4) $$t_{i}^{\mathrm{up}}=U\left(b_{i},f\right)=\frac{b_{i}}{r}$$

where *r* denotes the transmission rate of communication between the computing nodes, and $b_{i}$ is the amount of input data required to transmit the program execution of the computing task from the local user device to the computing node.

The upload delay of the task is divided into vehicle-to-vehicle transmission delay $t_{i}^{V2V}$ (V2V), vehicle-to-base station upload delay $t_{i}^{V2E}$ (V2E), transmission delay $t_{i}^{E2E}$ (E2E) between edge servers, and upload delay $t_{i}^{E2C}$ (E2C) from the edge server to the central cloud server.

(2) Cache delay: When the computational task $T_{i}$ offloaded to the ECS or CS at moment *t*, the task cache queuing time $t^{q}$ is defined as

(5) $$t_{i}^{q}=\sum_{j\in Q(t)}t_{j}^{\mathrm{exe}}$$

(3) Computation delay: Set f>0 (cycles/s) denotes the CPU computing capacity of the computing node. Therefore, the task computation delay $t_{i}^{\mathrm{exe}}$is

(6) $$t_{i}^{\mathrm{exe}\;}=J\left(c_{i},f\right)=\frac{c_{i}}{f}$$

Since the output data volume is usually much smaller than the input, and the data transmission rate of the downlink is much higher than that of the uplink, the transmission delay of the output is omitted in this model calculation, as also considered in [25,26,27]. There are four categories of computational processing described in this system, namely, local computation, service vehicle computation, edge server computation, and cloud server computation, where the binary variable $y_{i,j}=1$ denotes that the task vehicle ${\mathrm{TaV}}_{i}$ of the task $T_{i}$ is offloaded across the domain to the collaborative edge server computation. $y_{i,j}=0$ denotes edge computing within the edge computing domain. Then, the task $T_{i}$ of the total computation delay computed at $t_{i}$ is

(7) $$t_{i}=\left\{\begin{array}{cc} t_{i}^{\mathrm{exe}\;}, & a_{i,j}=0\\ t_{i}^{V2\;V}+t_{i}^{\mathrm{exe}\;}, & a_{i,j}=1\&j>K\\ t_{i}^{V2E}+t_{i}^{\mathrm{exe}}+t_{i}^{q}+y_{ij}\cdot t_{i}^{E2E}, & a_{i,j}=1\&0<j\leq K\\ t_{i}^{V2E}+t_{i}^{\mathrm{exe}}+t_{i}^{q}+t_{i}^{E2C}, & a_{i,j}=1\&j=0 \end{array}\right.$$

The total time delay for this collaborative computing system is

(8) $$t^{\mathrm{tol}}=\sum t_{i}$$

### 3.4. Energy Consumption Model

The main consideration is the task vehicle’s energy consumption in this system, which is divided into local calculation energy consumption and task offloading energy consumption. The energy consumption generated by the task vehicle ${\mathrm{TaV}}_{i}$ while it performs task $T_{i}$ locally, or the amount of the local computation energy consumption $e_{i}^{\mathrm{loc}\;}$, is

(9) $$e_{i}^{\mathrm{loc}\;}=p_{c}\cdot t_{i}^{\mathrm{exe}\;}$$

where $p_{c}$ indicates the power of the vehicle terminal’s CPU.

Task vehicles ${\mathrm{TaV}}_{i}$ perform task offloading to service computing nodes generated by transmission energy $e_{i}$, denoted by

(10) $$e_{i}^{\mathrm{up}}=\frac{p_{\mathrm{up}}\cdot t_{i}^{\mathrm{up}}}{\xi_{i}}$$

where $p_{\mathrm{up}}$ denotes that the vehicle terminal transmits power, and $\xi_{i}$ is the power amplifier’s efficiency of the task vehicle ${\mathrm{TaV}}_{i}$. In a general case, this system assumes that $\xi_{i}=1$, and the task vehicle ${\mathrm{TaV}}_{i}$ in the uplink energy consumption is calculated simply as $e_{i}^{\mathrm{up}}=p_{\mathrm{up}}\cdot t_{i}^{\mathrm{up}}$ [27]. The task $T_{i}$ of the task vehicle ${\mathrm{TaV}}_{i}$ is executed, and its generated energy consumption is

(11) $$e_{i}=\left\{\begin{array}{c} e_{i}^{\mathrm{loc}},a_{i,j}=0\\ e_{i}^{\mathrm{up}\;},\;\mathrm{others}\; \end{array}\right.$$

The total energy consumption of this collaborative computing system is

(12) $$e^{\mathrm{tol}}=\sum e_{i}$$

### 3.5. Prioritization Model

To improve task offloading utility, a comprehensive evaluation of computational task $T_{i}$ is performed based on task delay constraints and local computational urgency, and the priority of computational task $T_{i}$ offloading is determined. The model uses a mixed weighting approach to prioritize the computational task $T_{i}$, where priority $Pr_{i}$ is defined as

(13) $$\begin{array}{cc} \; & \begin{array}{c} Pr_{i}=\lambda_{1}\cdot W_{i}+\lambda_{2}\cdot U_{i} \end{array}\\ \; & W_{i}=\frac{1}{t_{i}^{\max}}\\ \; & U_{i}=\frac{1}{t_{i}^{\max}-t_{i}^{\mathrm{loc}}} \end{array}$$

where $\lambda_{1},\lambda_{2}\in \left[0,1\right]$ and satisfies $\lambda_{1}+\lambda_{2}=1$, and $U_{i}$ and $W_{i}$ are weighting factors. $W_{i}$ denotes the task value of the task $T_{i}$. $U_{i}$ denotes the computational task $T_{i}$ the task urgency, and $t_{i}^{\mathrm{loc}}$ is the local execution time of the task.

## 4. Computational Offloading Strategy Problem

For the cloud-edge-end collaborative computing system model in the Internet of Vehicles scenario proposed in Section 3, this section elaborates on the problem of the system task offloading strategy. In edge computing systems, the quality of service is mainly expressed in terms of the delay and energy consumption generated by the computational task completion. In the considered Internet of Vehicles scenario, this paper, considering both delay and energy consumption improvements, defines the task offloading utility of task vehicle ${\mathrm{TaV}}_{i}$ is defined as

(14) $$F_{i}=\delta_{i}^{t}\frac{t_{i}^{\mathrm{loc}}-t_{i}}{t_{i}^{\mathrm{loc}}}+\delta_{i}^{e}\frac{e_{i}^{\mathrm{loc}}-e_{i}}{e_{i}^{\mathrm{loc}}}$$

where $\delta_{i}^{t}$ is the time delay weight, $\delta_{i}^{e}$ denotes the energy consumption weight, and $\delta_{i}^{t}\;+\;\delta_{i}^{e}\;=\;1$, $\delta_{i}^{t},\delta_{i}^{e}\in \left[0,1\right]$, and i∈Ta. For example, a task vehicle ${\mathrm{TaV}}_{i}$ with a small battery capacity can increase $\delta_{i}^{e}$, decreasing $\delta_{i}^{t}$ and thus saving more energy at the cost of longer task delay. The task offloading utility of the system described is expressed as $\bar{F}=\left\{F_{i}|i\in \right.Ta\}$.

For a given offloading strategy *X*, the present collaborative computing system task offloading strategy problem is formulated as a problem of maximizing the offloading utility of the system, that is

(15) $$\begin{array}{cc} \; & \max\;\bar{F}\\ s.t. & C1:a_{i,j}\in \left\{0,1\right\},\forall i\in Ta,j\in N\\ \; & C2:\sum_{\begin{array}{c} j\in N \end{array}}a_{i,j}\leq 1,\forall i\in Ta\\ \; & C3:\sum_{\begin{array}{c} i\in Ta \end{array}}a_{i,j}\leq 1,\forall j\in \begin{array}{c} Se \end{array}\\ \; & C4:t_{i}\leq t_{i}^{\max},\forall i\in Ta\\ \; & C5:t_{i}\leq \left\{\begin{array}{c} \varphi_{i,j},\forall i\in Ta,\forall j\in Se\\ \varphi_{i}\;,\forall i\in Ta \end{array}\right.\\ \; & C6:d_{i,j}\leq R,\forall i\in Ta,\forall j\in Se \end{array}$$

where $\varphi_{i}$ denotes that ${\mathrm{TaV}}_{i}$ and BS can remain connected, $\varphi_{i,j}$ denotes that ${\mathrm{TaV}}_{i}$ and ${\mathrm{SeV}}_{j}$ can remain connected, and they are calculated as

(16) $$\varphi_{i}=\left\{\begin{array}{c} \left|\frac{L^{\prime }-x_{i}}{v_{i}}\right|,v_{i}>0\\ \left|\frac{-L^{\prime }-x_{i}}{v_{i}}\right|,v_{i}<0 \end{array}\right.$$

(17) $$\varphi_{i,j}=\left\{\begin{array}{c} 50,\;v_{i}=v_{j}\&\left|x_{i}-x_{j}\right|<R\\ \frac{R^{\prime }-\left(x_{i}-x_{j}\right)\mathrm{sign}\left(v_{i}-v_{j}\right)}{\left|v_{i}-v_{j}\right|},\;\mathrm{others}\; \end{array}\right.$$

where $L^{\prime }$ denotes that ${\mathrm{TaV}}_{i}$ can move a lateral distance within the communication range of V2I at a fixed transmission power, $R^{\prime }$ denotes that ${\mathrm{TaV}}_{i}$ can move a lateral distance within the communication range of V2V at a fixed transmission power, and sign(·) is a symbolic function, which is expressed in this equation as follows: when $v_{i}-v_{j}>0$, $\mathrm{sign}\left(v_{i}-v_{j}\right)=1$; when $v_{i}-v_{j}<0$, $\mathrm{sign}\left(v_{i}-v_{j}\right)=-1$. $\varphi_{i,j}=50$ denotes that the two vehicles ${\mathrm{TaV}}_{i}$ and ${\mathrm{SeV}}_{j}$ have the same speed and that the initial position is within the communication range, whereby the two vehicles can keep communication for a long time, thus assigning $\varphi_{i,j}$ to an enormous value. In other cases, when $\left(x_{i}-x_{j}\right)\mathrm{sign}\left(v_{i}-v_{j}\right)>0$, this indicates that ${\mathrm{TaV}}_{i}$ and ${\mathrm{SeV}}_{j}$ are moving away from each other; when $\left(x_{i}-x_{j}\right)\mathrm{sign}\left(v_{i}-v_{j}\right)<0$, this indicates that ${\mathrm{TaV}}_{i}$ and ${\mathrm{SeV}}_{j}$ are moving closer to each other.

The constraints in Equation (15) are explained as follows: constraints C1 and C2 imply that each task can be executed locally or offloaded to at most one service computing node; constraint C3 implies that each service vehicle can service at most one task vehicle; constraint C4 specifies that each task must be completed within the specified maximum time delay limit; constraint C5 specifies that the task offloaded to the service vehicle must be completed within the two-vehicle maintain-communication time, or that offloading to the ECS must be completed within the hold-communication time with the BS; constraint C6 specifies that the straight-line distance $d_{i,j}$ between the task vehicle and the service vehicle for both vehicles must be no greater than the communication distance *R* for the task to be offloaded.

## 5. Multilateral Collaborative Computing Offloading Strategy Based on the M-TSA Algorithm

To cope with the more complex cloud-edge-end collaborative computing system in the Internet of Vehicles scenario, this section proposes a multi-strategy collaboration-based TSA algorithm (M-TSA) and then proposes a multilateral collaborative computing offload strategy based on the M-TSA algorithm. The M-TSA algorithm, which introduces multiple population evolution strategies into the TSA algorithm, can better meet the optimization of the computational offload quality of service metrics (system delay, system energy consumption) in the collaborative computing system in the Internet of Vehicles scenario.

### 5.1. Standard TSA Algorithm

The Tunicate Swarm Algorithm (TSA) is an intelligent swarm optimization algorithm proposed by Kaur et al. [28] to simulate the foraging behavior of a swarm of animals in the ocean. Its execution includes jet propulsion and group behavior. It has the advantages of a simple structure, strong local search ability, high accuracy of search and optimization, and has been validated in function optimization problems and engineering applications. However, the search mode is single and there is no individual memory, so the local search is not sufficient and the accuracy is low when solving high-complexity problems.

#### 5.1.1. Jet Propulsion

Equation (18) denotes the principle of conflict avoidance between individuals, *A* denotes the factor of conflict avoidance between individuals, *G* is gravity, and $c_{1},c_{2},c_{3}$ is a random number between [0, 1], respectively. *H* represents the social interaction between individuals, and $p_{\min},\;p_{\max}$ are the initial and subordinate velocities of social interactions between individuals, respectively, setting $p_{\min}=1,\;p_{\max}=4$. Equation (19) denotes the movement toward the optimal individual, PD denotes the distance between the food (optimal individual) and the individual, *k* is the current iteration number, FS is the position of food, and $P_{p}\left(k\right)$ denotes the current position of the individual. Equation (20) denotes convergence to the optimal individual, and $r_{\mathrm{and}}$ is a random number between [0, 1].

(18) $$\left\{\begin{array}{c} \begin{array}{c} A=\frac{G}{H} \end{array}\\ \begin{array}{c} G=c_{2}+c_{3}-2c_{1} \end{array}\\ \begin{array}{c} H=\left\lfloor p_{\min}+c_{1}\left(p_{\max}-p_{\min}\right)\right\rfloor \end{array} \end{array}\right.$$

(19) $$\begin{array}{c} PD=FS-r_{\mathrm{and}}\cdot P_{p}\left(k\right) \end{array}$$

(20) $$\begin{array}{c} P_{p}\left(k\right) \end{array}=\left\{\begin{array}{c} FS+A\cdot PD,\;r_{\mathrm{and}}\geq 0.5\\ FS-A\cdot PD,\;r_{\mathrm{and}}<0.5 \end{array}\right.$$

#### 5.1.2. Swarm Behavior

Equation (21) represents the location of the optimal solution of the updated individual, which is calculated based on the optimal location of the current two generations of search individuals, and the tunicate individuals perform swarm behavior to gather towards the food’s (the optimal individual’s) location.

(21) $$P_{p}\left(k+1\right)=\frac{P_{p}\left(k\right)+P_{p}\left(k+1\right)}{2+c_{1}}$$

### 5.2. M-TSA Algorithm

For the complex computational offloading problem of the more complex cloud-edge-end collaborative computing system in the vehicle networking scenario, the M-TSA algorithm is proposed to improve the algorithm’s global exploration and local exploitation capabilities by introducing a memory learning strategy, a Levy flight strategy, and an adaptive dynamic weighting strategy based on the standard encapsulated swarm algorithm, as described below.

#### 5.2.1. Memory Learning Strategy

The memory learning strategy, introduced by Particle Swarm Optimization (PSO) memory learning, includes the update speed *v* and position *x*, as

(22) $$\begin{array}{c} v_{i}=\omega \times v_{i}+c_{1}\times r_{\mathrm{and}}\times \left(pbest_{i}-X_{i}\right)+\\ c_{2}\times r_{\mathrm{and}\;}\times \left(gbest-X_{i}\right)\\ X_{i}=X_{i}+v_{i}\\ \omega \left(t\right)=\omega_{\min}+\left(\omega_{\max}-\omega_{\min}\right)\left(K-k\right)/K \end{array}$$

where $v_{i}$ is the individual velocity, $X_{i}$ is the individual position, pbesti is the individual optimal solution, gbest is the global optimal solution, ω is the inertia factor, $c_{1}$ is the self-learning factor, and $c_{2}$ is the swarm learning factor. The memory learning of PSO is introduced in this algorithm to strengthen the self-memory learning; let $c_{1}=2$, $c_{2}=2$, $r_{\mathrm{and}}$ is the random number between [0, 1], *k* is the current population iteration number, *K* is the maximum iteration number, and it is taken in this algorithm $\omega_{\max}=0.9$, $\omega_{\min}\;=\;0.4$. The dynamic inertia factor ω has better merit-seeking results than fixed values, so this algorithm adopts a linearly decreasing weight strategy, with the iteration period ω decreasing gradually, and the swarm individuals have strong global search merit-seeking ability in the early stage and enhanced local search merit-seeking ability in the later stage.

#### 5.2.2. Levy Flight Strategy

To increase the diversity of populations, this algorithm introduces a stochastic cross-learning strategy based on Levy flight, which allows the algorithm to have greater randomness in the optimization process and avoid the algorithm from falling into the local optimum.

(23) $$X_{i}\left(k+1\right)=\left\{\begin{array}{c} X_{i}\left(k\right)+\alpha \left(X_{j}\left(k\right)\cdot \mathrm{lev}y\left(\beta \right)-X_{i}\left(k\right)\right),r_{\mathrm{and}}<J\\ X_{i}\left(k\right)+\alpha \left(X_{j}\left(k\right)-X_{i}\left(k\right)\cdot \mathrm{levy}\left(\beta \right)\right),J\leq r_{\mathrm{and}\;}<1-J\\ X_{i}\left(k\right)+\alpha \left(X_{j}\left(k\right)-X_{i}\left(k\right)\right)\cdot \mathrm{levy}\left(\beta \right),r_{\mathrm{and}}\geq 1-J \end{array}\right.$$

(24) $$\mathrm{levy}\left(\beta \right)=\frac{u}{|\begin{array}{c} s \end{array}{|}^{-\beta }}$$

(25) $$\sigma_{u}={\left[\frac{\Gamma \left(1+\beta \right)\sin\left(\frac{\pi \beta }{2}\right)}{\Gamma \left(\frac{1+\beta }{2}\right)\beta \times 2^{\frac{\beta -1}{2}}}\right]}^{\frac{1}{\beta }},\sigma_{\begin{array}{c} s \end{array}}=1$$

where $u∼N\left(0,\sigma_{u}\right)$, $s∼N\left(0,\sigma_{s}\right)$, $r_{\mathrm{and}}$ is a random number between [0, 1], and J∈[0,1] denotes the probability variable that determines which cross-learning method is used by the individuals in the population. In order for individuals in the population to select each cross-learning mode with equal probability, set J=1/3. α denotes the cross-learning coefficient;levy(·) denotes the random number that satisfies the Levy distribution.

#### 5.2.3. Adaptive Dynamic Weighting Strategy

To improve the performance of the TSA algorithm, an adaptive dynamic weighting strategy is proposed to balance the global exploration and local exploitation capabilities of the TSA algorithm. For the position of each capsule individual, we use the following equation to enhance the algorithm’s ability to search for the global optimum and increase the current capsule search step to enhance the algorithm’s ability to escape the extreme values, calculated as follows.

(26) $$\begin{array}{c} P_{p}\left(k\right)=\left\{\begin{array}{c} FS+2\cdot A\cdot PD,\;r_{\mathrm{and}\;}\geq 0.5\\ FS-A\cdot PD,\;r_{\mathrm{and}\;}<0.5 \end{array}\right. \end{array}$$

where *A* is the conflict avoidance factor between individuals, PD is the distance between the food (optimal individual) and the individual, *k* is the current iteration number, FS is the position of the food, $P_{p}\left(k\right)$ denotes the position of the current individual, and $r_{and}$ is a random number between [0, 1].

To balance the global exploration and local exploitation abilities of the capsule swarm algorithm, this paper proposes an adaptive dynamic weighting strategy to update the positions of capsule individuals. In this strategy, the updated formula for the position of the capsule individual includes the current position of the individual, the position of the previous generation individual, and the adaptive weight. The size of the adaptive weight is related to the position of the capsule individual and can be dynamically adjusted during the iteration of the algorithm. When the adaptive weight is larger, the step size of the individual position update is smaller, which is beneficial to the global exploration ability of the algorithm. When the adaptive weight is smaller, the step size of the individual position update is larger, which is beneficial to the local exploitation ability of the algorithm. Compared with the random parameters in the original TSA algorithm, the adaptive dynamic weighting strategy can improve the performance of the algorithm and avoid the problems caused by the blindness of the algorithm. The specific calculation formula is shown as follows:

(27) $$z=2e^{-(3k/K)}$$

where *k* is the number of current iterations and *K* is the maximum number of iterations.

The swarm behavior update formula for introducing adaptive dynamic weight values in the swarm behavior of the M-TSA algorithm is

(28) $$\begin{array}{c} P_{p}\left(k+1\right)=\frac{P_{p}\left(k\right)+P_{p}\left(k+1\right)}{2+z} \end{array}$$

where $P_{p}\left(k\right)$ denotes the current individual’s position, and *z* denotes the adaptive dynamic weight value. During the iteration of the algorithm, the adaptive weight value decreases gradually with time, which leads to an overall increase in the position update weight and a corresponding increase in the update step size, which makes the algorithm a strong exploration capability at a later stage.

#### 5.2.4. Adaptive Dynamic Regulation of Populations

This algorithm performs adaptive dynamic adjustment of the number of individuals performing memory cross-learning and jet propulsion to enhance the ability of global full search finding of the population. In the early iteration, most of the individuals in the population of this algorithm performed memory cross-learning to increase the population’s local search and enhance the global search directionality in the later iteration. In the later iteration, to avoid falling into the local optimal results, most of the individuals performed TSA jet propulsion mode to improve the algorithm’s ability to jump out of the local for global search, effectively balancing the local search and global search abilities. The algorithm uses an adaptive decay adjustment strategy for the number of subgroup individuals num, as defined below.

(29) $$\begin{array}{c} num\left(k\right)=S\cdot e^{-3k/K}-1 \end{array}$$

where *k* is the number of current population iterations, *K* is the maximum number of iterations, and *S* is the overall population number of individuals. The variable gbest(k) is used to denote the global optimal individual at generation *k*. The steps of the population adaptive dynamic adjustment algorithm are shown in Algorithm 1.    
**Algorithm 1** Adaptive dynamic regulation of populations algorithm.**Input:** S,k,K,gbest**Output:** num**procedure** Anum**if** gbest(k+1)=gbest(k) **then** /* gbest(k) is the global optimal individual at generation *k*.*/      num←0**else**      num←S×exp(−3×k/K)−1**end if****return** num**end procedure**

#### 5.2.5. M-TSA Algorithm Steps

**Figure 3:**
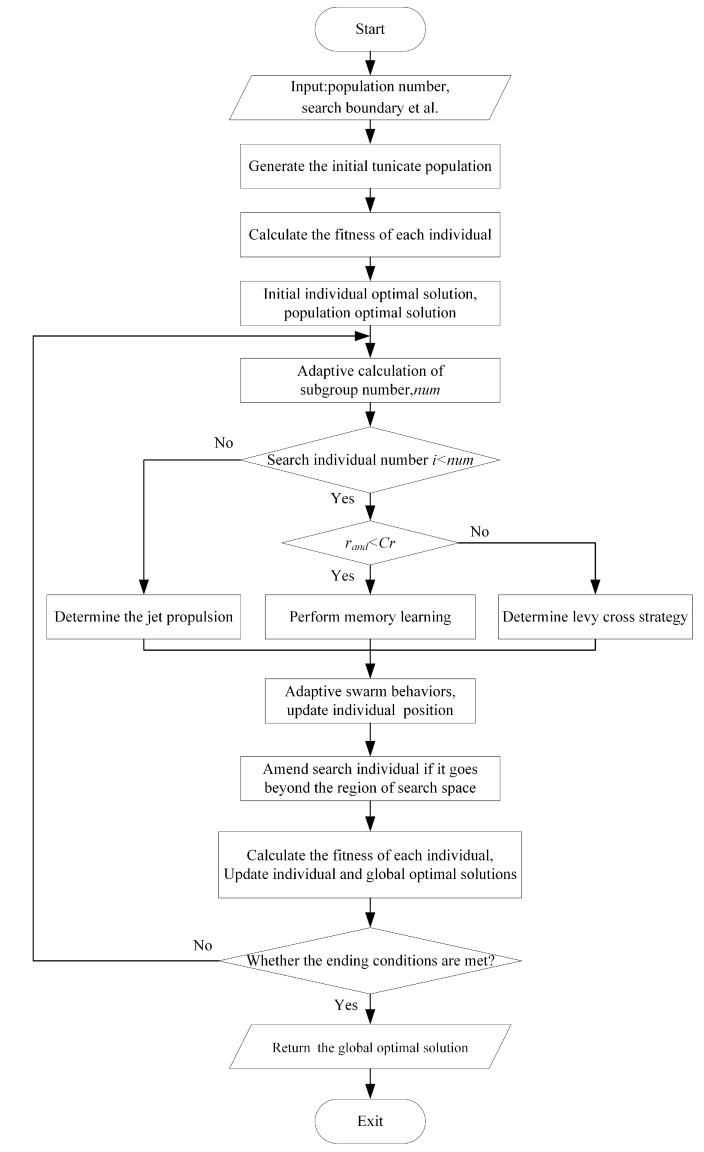
M-TSA algorithm steps.

The flow of the M-TSA algorithm is shown in Figure 3, and the specific steps are as follows. The M-TSA algorithm pseudocode is shown in Algorithm 2.
**Algorithm 2** M-TSA algorithm.**Input:** S,K, populationX**Output:** $X_{\mathrm{best}}$**procedure** M-TSA$p_{\min}\leftarrow 1$$p_{\max}\leftarrow 3$X1←0$pbest,\;X_{\mathrm{best}}\leftarrow$CaculateFitness(*X*)/* Initialize the individual fitness value using the CalculateFitness function.*/**for** k←1toK **do**      num← Anum( $S,k,K,X_{\mathrm{best}}$ ) /* Anum() is adaptive dynamic regulation of populations algorithm, see Algorithm 1.*/      **for** i←1toS **do**            **if** i<num **then**                  $r_{\mathrm{and}}\leftarrow$ Rand() /* Rand() is a function to generate the random number in the range [0, 1]. */                  **if** $r_{\mathrm{and}}<Cr$ **then**                        X1←X+v /*Memory learning strategy according to Equation (22).                        */                  **else**                        X1← Levy_strategy() /* Levy_strategy() is a function that Levy flight strategy according to Equation (23).*/                  **end if**            **else** /*Jet propulsion according to Equations (18), (19), (26).*/                  $c_{1},c_{2},c_{3}\leftarrow$ Rand()                  $H\leftarrow \left\lfloor p_{\min}+c_{1}\left(p_{\max}-p_{\min}\right)\right\rfloor$                  $A\leftarrow \left(c_{2}+c_{3}\right)-2\times c_{1}/H$                  $PD\leftarrow \mathrm{abs}(X_{\mathrm{best}}-\mathrm{Rand}()\times X)$                  **if** Rand()>=0.5 **then**                        $X1\leftarrow X_{\mathrm{best}}+2*A*PD$                  **else**                        $X1\leftarrow X_{\mathrm{best}}-A*PD$                  **end if**            **end if**            /*Adaptive swarm behavior to Equation (28).*/            X←(X1+X)/(2+z)      **end for**      $pbest,X_{\mathrm{best}}\leftarrow$CaculateFitness(*X*) /* Calculate the individual fitness value of the new population using CaculateFitness function*/**end for****return**$X_{\mathrm{best}}$**end procedure** **procedure** CaculateFitness(*X*)      **for** i←1toS **do**            fit[i]←fitnessfun(X[i,:]) /* Calculate the fitness of each individual */            **if** fit[i]<pbestfit[i] **then**                  pbestfit[i]←fit[i]                  pbest[i]←X[i]            **end if**      **end for**      **if** $Min\left(pbestfit\right)<Xfit_{\mathrm{best}}$ **then**            $X_{\mathrm{best}}\leftarrow X\left[argmin\left(pbestfit\right)\right]$/* argmin() is the function to obtain the minimum value index */      **end if****return** $pbest,X_{\mathrm{best}}$**end procedure**

Step 1: First, according to the task example, randomly generate the initial population of the capsule; the individuals in the population include the location to be optimized $x_{i}\left(i=1,2,\ldots ,n\right)$ and its fitness value to be optimized $f_{i}$.

Step 2: Calculate the individual fitness value $f_{i}$, and derive the initial per-individual optimal solution $pbest_{i}$ and the global optimal solution gbest.

Step 3: Based on the update situation of the global optimal individual gbest and the number of iterations, the number of subpopulation individuals num is calculated adaptively, as described in Algorithm 1.

Step 4: The num individuals of the subpopulation perform memory learning or Levy flight strategy, when the random number $r_{and}<Cr$, performing memory learning, Cr is the cross-learning factor; the remaining S−num individuals perform the jet propulsion of TSA, where convergence to the optimal individual is calculated according to the improved Equation (26).

Step 5: Apply the adaptive dynamic weighting strategy to perform swarm behavior learning according to Equation (28), update its position, and generate a new generation of population.

Step 6: New populations are checked for transgression, and individuals beyond the constraint range are processed for transgression.

Step 7: Calculate the individual fitness value of the new population, and update the individual optimal solution $pbest_{i}$ and the global optimal solution gbest.

Step 8: Determine whether the maximum number of iterations is reached, and if it is satisfied, output the global optimal solution gbest; otherwise, return to Step 2.

### 5.3. Multilateral Collaborative Computing Offloading Strategy Based on the M-TSA Algorithm

#### 5.3.1. Computational Offloading Strategy’s Code

To facilitate the task offloading strategy execution in the swarm intelligence optimization algorithm, the computational nodes are encoded. This offloading strategy *X* is defined as $X=\left\{x_{1},x_{2},\ldots ,x_{i},\ldots ,x_{I}\right\}$, where $x_{i}$ is the task vehicle ${\mathrm{TaV}}_{i}$’s task computation node $x_{i}\in \left\{0,1,2,\ldots m+1,\ldots ,M+1,M+2,\ldots M+j_{m}+1,\ldots ,M+J_{m}+1\right\}$, whereby $x_{i}=0$ denotes the task vehicle ${\mathrm{TaV}}_{i}$ local compute node, $x_{i}=1$ indicates at the central cloud server compute node, $x_{i}=\left\{2,\ldots m+1,\ldots ,M+1\right\}$ indicates at the edge server compute node, and $x_{i}\geq M+2$ when $x_{i}=M+j_{m}+1$ indicates at the service vehicle ${\mathrm{SeV}}_{j}$ computing node.

**Figure 4:**
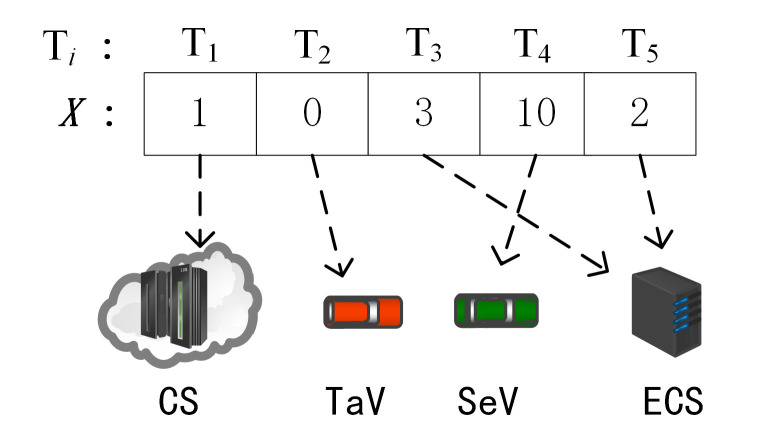
Computational offloading strategy’s code.

The offload policy is coded as shown in Figure 4, assuming that the task set $T_{i}\;=\;\left\{T_{1},T_{2},T_{3},T_{4},T_{5}\right\}$, M=4 means there are four edge server ECS, and the offloading strategy X={1,0,3,10,2} indicates that $T_{1}$ is offloaded to CS for execution, $T_{2}$ is executed locally, $T_{3}$ is offloaded to ${\mathrm{ECS}}_{2}$ execution, $T_{4}$ is offloaded to ${\mathrm{SeV}}_{5}$ for execution, and $T_{5}$ is offloaded to ${\mathrm{ECS}}_{1}$.

#### 5.3.2. Computational Offloading Strategy’s Code

In the TSA algorithm, the fitness function is used to evaluate the distance between the tunicate individual and the food source, i.e., the gap between this solution and the optimal solution to the problem. This offloading strategy is evaluated in three aspects, namely, computational delay, energy consumption, and offloading utility. The fitness evaluation function is constructed with the offloading utility of balanced computational delay and energy consumption, and the fitness evaluation value for the offloading strategy *X* is f(X), as shown in the following equation:

(30) $$f\left(X\right)=\bar{F}$$

where *F* denotes the system task offloading utility, $F=\left\{F_{i}|i\in Ta\right\}$.

#### 5.3.3. M-TSA Based Multilateral Collaborative Computing Offloading Strategy’s Algorithm Steps

For the complex computational offloading problem in the cloud-edge-end collaborative computing system in the Internet of Vehicles scenario, multiple evolutionary strategies are introduced, and a multilateral collaborative computational offloading strategy based on the M-TSA algorithm is proposed with the following algorithmic steps:

Step 1: Create cloud-edge-end collaborative computing system task instances in the Internet of Vehicles scenario, including creating CS instances, edge computing group Es, task vehicle set Ta, and service vehicle set Se that simulate real road conditions and vehicle movement.

Step 2: According to Equation (13), calculate the task offloading priority of each task $T_{i}$ in each edge computing domain and determine the task offloading order from the highest priority to the lowest priority.

Step 3: According to Equation (15) constraints C4–C6, predict the set of task unloadable nodes SeN, i.e., the individual boundary of the population, to narrow the search range of the algorithm and improve the task unload utility.

Step 4: Execute the M-TSA algorithm, see Section 5.2.5 for details. Take the ordered task set Ta and the predicted node set SeN as inputs, and execute the M-TSA algorithm to derive the optimal computational offloading decision $X_{\mathrm{best}}$.

## 6. Simulation Verification

To verify the effectiveness of the proposed M-TSA-based multilateral collaborative computing offloading strategy, this section presents our simulation experiments using Python, and the main parameters of the experiments are shown in Table 2. The experiments are conducted to compare three computing systems, namely, cloud-edge-end collaborative computing, end-edge collaborative computing, and local computing. Further, the experiments are conducted to compare the offloading strategies based on the M-TSA algorithm with the TSA algorithm, PSO algorithm, Grey Wolf Optimizer algorithm (GWO), and Differential Evolution Algorithm (DE) offloading strategy comparison experiments. The algorithm parameters of this experiment are set as iteration number K=50, population size size=40, one central cloud server, four edge computing domains, and a random group of vehicles in one computational domain.

### 6.1. Cloud-Edge-End Architecture Verification

Under the same experimental environment and the same task instance, the mixed weights of delay and energy consumption ($\delta_{i}^{t}=0.8,\delta_{i}^{e}=0.2$) under the M-TSA algorithm are compared, and the optimization results of cloud-edge-end collaborative computing, end-edge collaborative computing, and local computing for three computing systems on delay and energy consumption are discussed. In the created task instance, the three computing systems are run 20 times independently, and the average of the optimal solutions of the results of 20 runs of each algorithm is taken.

**Figure 5:**
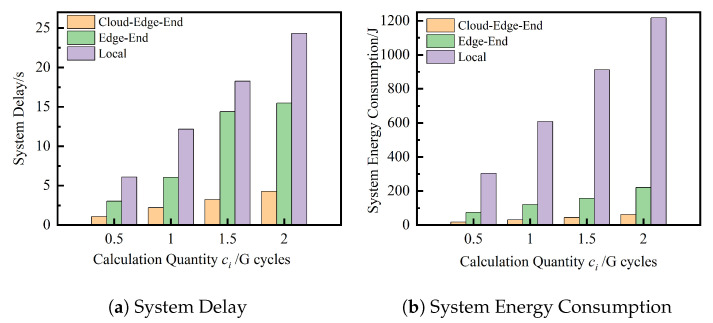
Comparison of different computing systems.

As can be seen in Figure 5, in the same experimental environment and with the same task instance input, the solution results of the cloud-edge-end collaborative computing system under the mixed weight evaluation are significantly better than those of the other two architectural computing systems, resulting in a smaller system delay and lower system energy consumption, and the advantage grows as the task computation volume increases. The experimental results show that the cloud-edge-end collaborative computing system is significantly better than other architectures and can realize complementary resources of cloud computing, edge nodes, and vehicle terminal devices, which can be flexibly configured according to the characteristics of the task and real-time demand to better adapt to different task scales.

### 6.2. Delay and Energy Mixing Weighting Optimization Comparison

**Figure 6:**
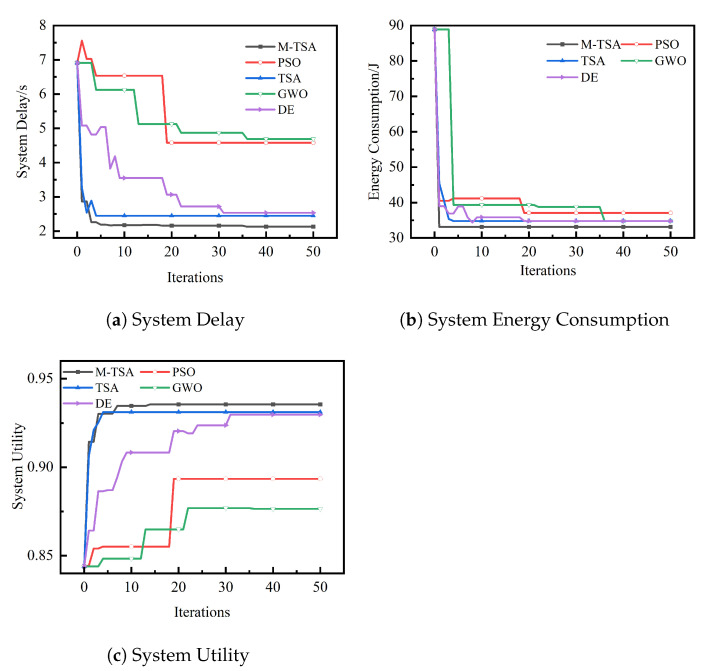
When $\delta_{i}^{t}=0.8,\delta_{i}^{e}=0.2$, the comparison curve of system utility and delay and energy consumption calculated by different algorithms.

Figure 6 depicts the optimization comparison results of offloading utility, delay, and energy consumption for five algorithms to calculate offloading under mixed weights of delay and energy consumption with the same experimental environment, the same task instance, and the same initial population when the number of TaV is 20 and the number of SeV is 30. In the created task instance, the five algorithms are run 20 times independently with the same input, and the optimal solution is taken from the results of the 20 runs of each algorithm.

As can be seen in Figure 6, the solution results of the M-TSA algorithm under the mixed weight evaluation are significantly better than the other four algorithms in the same experimental environment with the same task instances and the same initial population input, obtaining higher offloading utility, a shorter system time delay, and a lower system energy consumption, which is proof that the M-TSA algorithm has a stronger global optimization-seeking ability to derive the optimal computational offloading strategy. In addition, it can be seen in Figure 6 that the M-TSA algorithm can obtain the optimal solution in fewer iterations compared to other algorithms, indicating that the M-TSA algorithm has a fast optimality finding capability, which enhances its application to delay-sensitive vehicular networking special scenarios to compute offloading strategies.

### 6.3. Delay Orientation Optimization Test

**Figure 7:**
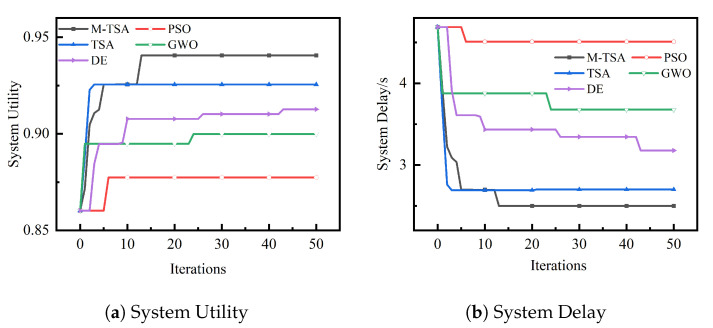
Comparison curve of system utility and delay of different algorithms for computational offloading in delay orientation optimization.

Figure 7 depicts the comparison results of offloading utility and delay for five algorithms to perform delay orientation optimization ($\delta_{i}^{t}=1.0,\delta_{i}^{e}=0.0$) experiments with the same experimental environment, the same task instances, and the same initial population when the number of TaV is 20 and the number of SeV is 30.

From Figure 7, it can be seen that the M-TSA proposed also has better results in calculating the offloading directed optimization delay compared with the PSO, TSA, GWO, and DE algorithms. From Figure 7a, we can see that the M-TSA algorithm has several large upward jumps relative to other algorithms, which in turn leads to better solutions. This is proof that the M-TSA algorithm has a stronger ability to jump out of the local global optimum and can continuously jump out of the local to fully search the global to arrive at the optimal computational offloading strategy.

### 6.4. Impact of Changes in the Number of Task Vehicles in the Computational Domain

This section is a simulation experiment in which the number of service vehicles (SeV) is 30, given $\delta_{i}^{t}=0.8,\delta_{i}^{e}=0.2$, and the optimization comparison results of offloading utility, time delay, and energy consumption for five algorithms for the different number of task vehicles in the same experimental environment and same task instance are presented. The five algorithms are run 20 times independently with the same input under a fixed number of TaV, and the average of the optimal solutions of the 20 runs of each algorithm is taken. Please refer to Table 3, Table 4 and Table 5 for the data on the impact of the number of TaV.

**Figure 8:**
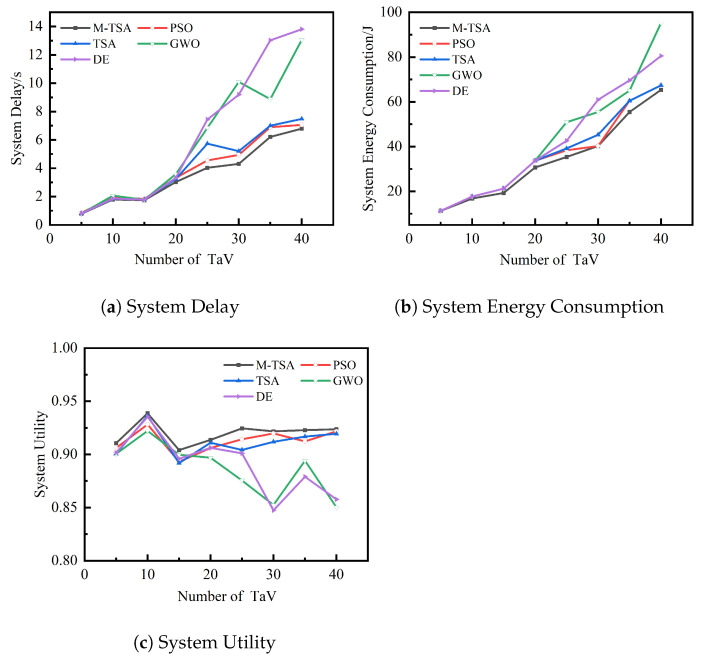
Effect of number of TaV on system utility, delay, and energy consumption.

As can be seen in Figure 8, the solution results of the M-TSA algorithm proposed are significantly better than the other four algorithms for the different number of task vehicles. It can derive a better computational offloading strategy, which enables the vehicle cooperative system to handle all computational tasks with higher offloading utility, a minimum system time delay, and a minimum system energy consumption, indicating that this algorithm is effective.

## 7. Conclusions

We discuss the problem of simultaneous computational offloading of multiple vehicles on a two-way straight highway in an Internet of Vehicles scenario and design a vehicle computational network model based on cloud-edge-end collaboration. The offloading utility, system time delay, and system energy consumption are the optimization objectives, and the vehicle motion characteristics and task time delay sensitivity are taken into account to make the computational offloading scheme more consistent with the actual, real situation. The simulation results show that the proposed offloading strategy can significantly improve the system task offloading utility and effectively reduce the system time delay and system energy consumption. In future research, relevant strategies will be further designed for more complex Internet of Vehicles scenarios to better match the actual situation.

The proposed approach takes into account the vehicle motion characteristics and task delay sensitivity to make the computational offloading scheme more realistic, but further challenges such as dynamic changes in the vehicle network topology and unreliable connections still need to be addressed. An interesting future research direction for this work is to employ predictive algorithms to predict the location and connectivity of vehicles for more accurate design and tuning of computational offloading strategies to better match the real-world situation.

## Figures and Tables

**Table 1:** Meaning of parameters.

Parameters	Meaning
TaV	The task vehicles.
SeV	The service vehicles.
*v*	The vehicle speed.
$f_{i}$	The CPU computing capacity of TaV.
$f_{j}$	The CPU computing capacity of SeV.
$f_{c}$	The CPU computing capacity of CS.
$f_{s}$	The CPU computing capacity of ECS.
$f_{ns}$	The CPU computing capacity of other collaborative computing ECSs.
*h*	The linear distance between BS and the road.
*L*	The communication coverage radius of BS.
*R*	The communication distances of V2V.
$r^{V2I}$	The transmission rate of V2I.
$r^{V2V}$	The transmission rate of V2V.
$r^{E2E}$	The transmission rate of E2E.
$r^{E2C}$	The transmission rate of E2C.
$p_{\mathrm{up}}$	The vehicle terminal transmits power,
$p_{c}$	The power of the vehicle terminal’s CPU.
$b_{i}$	The amount of data required to complete the task.
$c_{i}$	The amount of computation to complete the task.
$t_{i}^{\max}$	The maximum delay limit of the computation task.

**Table 2:** Parameters of the experiments.

Parameters	Value
*v*/km$\cdot h^{-1}$	[30, 80]
$f_{i}$/GHz	[0.5, 1]
$f_{j}$/GHz	[1, 2]
$f_{c}$/GHz	50
$f_{s}$/GHz	20
$f_{ns}$/GHz	[10, 20]
*h*/m	1
*L*/m	1000
*R*/m	20
$r^{V2I}$/Mbit$\cdot s^{-1}$	1000
$r^{V2V}$/Mbit$\cdot s^{-1}$	400
$r^{E2E}$/Mbit$\cdot s^{-1}$	2000
$r^{E2C}$/Mbit$\cdot s^{-1}$	1500
$p_{\mathrm{up}}$/W	30
$p_{c}$/W	50
$b_{i}$/Mbit	[10, 100]
$c_{i}$/G cycles	[0.1, 2]
$t_{i}^{\max}$/s	[0.2, 5]

The communication simulation parameters of V2I and V2V are set with reference to the 5G-V2X network standard adopted by most car companies nowadays.

**Table 3:** Data on the impact of the number of TaV—System Delay.

Number of TaV	System Delay/s				
	M-TSA	PSO	TSA	GWO	DE
5	0.7816	0.8615	0.8075	0.8075	0.8037
10	1.7947	1.9322	1.8426	2.0618	1.8569
15	1.7502	1.8346	1.7969	1.7739	1.7800
20	3.0281	3.3211	3.2356	3.5881	3.3168
25	4.0265	4.5479	5.7373	6.8484	7.4391
30	4.3004	4.9402	5.2001	10.0968	9.1898
35	6.2045	6.8987	7.0080	8.8794	13.0259
40	6.7945	7.0596	7.4746	13.0574	13.8094

**Table 4:** Data on the impact of the number of TaV—System Energy Consumption.

Number of TaV	System Energy Consumption/J				
	M-TSA	PSO	TSA	GWO	DE
5	11.28	11.28	11.28	11.28	11.28
10	16.82	17.67	17.67	17.67	17.67
15	19.24	21.24	21.24	21.24	21.24
20	30.69	33.69	33.69	33.69	33.69
25	35.37	38.37	39.14	50.88	42.56
30	40.26	40.26	45.26	55.44	60.93
35	55.45	60.45	60.45	65.00	69.59
40	65.35	67.35	67.35	95.26	80.54

**Table 5:** Data on the impact of the number of TaV—System Utility.

Number of TaV	System Utility				
	M-TSA	PSO	TSA	GWO	DE
5	0.9106	0.9061	0.9006	0.9005	0.9016
10	0.9387	0.9282	0.9362	0.9221	0.9354
15	0.9041	0.8930	0.8918	0.8997	0.8960
20	0.9137	0.9061	0.9110	0.8969	0.9062
25	0.9245	0.9142	0.9042	0.8754	0.9009
30	0.9218	0.9198	0.9118	0.8524	0.8473
35	0.9228	0.9122	0.9167	0.8941	0.8789
40	0.9236	0.9217	0.9195	0.8502	0.8576

## Data Availability

Not applicable.
